# Emerging Significance of Ginsenosides as Potentially Reversal Agents of Chemoresistance in Cancer Therapy

**DOI:** 10.3389/fphar.2021.720474

**Published:** 2021-12-17

**Authors:** Jin-Feng Xu, Yan Wan, Fei Tang, Lu Chen, Yu Yang, Jia Xia, Jiao-Jiao Wu, Hui Ao, Cheng Peng

**Affiliations:** ^1^ State Key Laboratory of Characteristic Chinese Medicine Resources in Southwest China, Pharmacy College, Chengdu University of Traditional Chinese Medicine, Chengdu, China; ^2^ Innovative Institute of Chinese Medicine and Pharmacy, Chengdu University of Traditional Chinese Medicine, Chengdu, China

**Keywords:** ginsenosides, reverse, chemoresistance, chemotherapy, cancer

## Abstract

Chemoresistance has become a prevalent phenomenon in cancer therapy, which alleviates the effect of chemotherapy and makes it difficult to break the bottleneck of the survival rate of tumor patients. Current approaches for reversing chemoresistance are poorly effective and may cause numerous new problems. Therefore, it is urgent to develop novel and efficient drugs derived from natural non-toxic compounds for the reversal of chemoresistance. Researches *in vivo* and *in vitro* suggest that ginsenosides are undoubtedly low-toxic and effective options for the reversal of chemoresistance. The underlying mechanism of reversal of chemoresistance is correlated with inhibition of drug transporters, induction of apoptosis, and modulation of the tumor microenvironment(TME), as well as the modulation of signaling pathways, such as nuclear factor erythroid-2 related factor 2 (NRF2)/AKT, lncRNA cancer susceptibility candidate 2(CASC2)/ protein tyrosine phosphatase gene (PTEN), AKT/ sirtuin1(SIRT1), epidermal growth factor receptor (EGFR)/ phosphatidylinositol 3-kinase (PI3K)/AKT, PI3K/AKT/ mammalian target of rapamycin(mTOR) and nuclear factor-*κ*B (NF-*κ*B). Since the effects and the mechanisms of ginsenosides on chemoresistance reversal have not yet been reviewed, this review summarized comprehensively experimental data *in vivo* and *in vitro* to elucidate the functional roles of ginsenosides in chemoresistance reversal and shed light on the future research of ginsenosides.

## 1 Introduction

Chemoresistance is regarded as the capability of cancer cells to evade or to cope with the presence of therapeutics, and a vital challenge that researchers manage to reverse. Chemoresistance occurs before treatment and is acquired after therapy, which causes disease relapse and metastasis and worsens the clinical outcome of the cancer patients in most cases (Gottesman et al., 2016; Hamed et al., 2019). As reported, more than 90% of cancer patients died from metastatic cancer in different degrees, which was related to chemoresistance to varying degrees (Wilson et al., 2009). Notably, occurrence of chemoresistance is attributed to multiple targets and pathways, including inhibition in drug transporter proteins expression and membrane fluidity, alteration in the drug target, apoptosis induction, and modulation of autophagy. Mechanically, the occurrence of multiple factors such as drug efflux, apoptosis, autophagy and microenvironment, are related to multiple pathways and targets, including NRF2/AKT, CASC2/PTEN, AKT/SIRT1, EGFR/PI3K/AKT, PI3K/AKT/mTOR, and NF-*κ*B (Grossman and Altieri, 2001; Vadlapatla et al., 2013). In recent years, chemoresistance reversal has attracted attention worldwide but is still in a bottleneck period. Until now, three measures including chemical drugs, biological therapy and hyperthermia are taken to reverse chemoresistance. Unfortunately, these methods are not suitable for clinical promotion because of their single target and function property, serious side effects, expensive cost, and high technical requirements. Furthermore, these strategies have no anti-cancer effects themselves, but bring new problems to cancer therapy (Baird and Kaye, 2003). Thus, better therapeutic approaches with multiple targets, multi-functions, higher efficiency, and lower cost and toxicity are needed to overcome chemoresistance.

**FIGURE 1:**
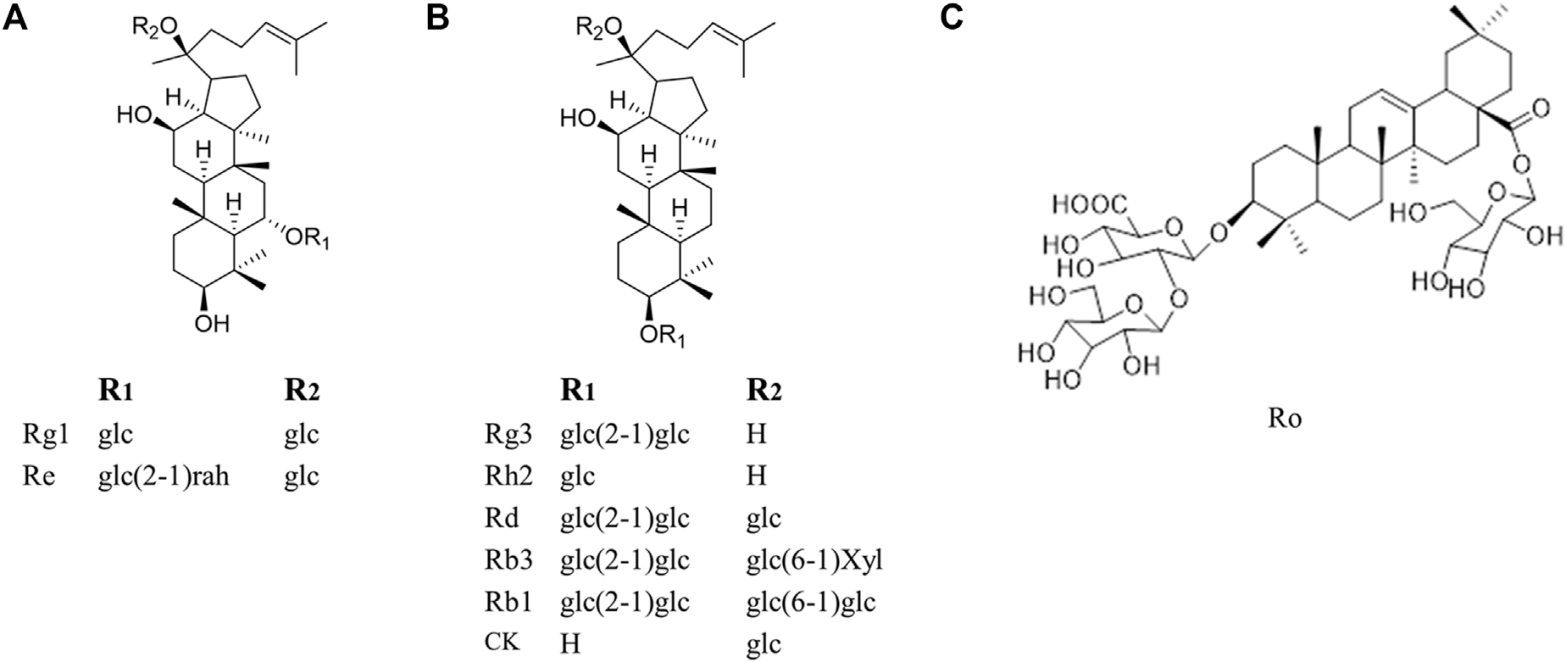
Molecular structures of representative ginsenosides separated from ginseng in our study. **(A)** Protopanaxatriol ginsenosides **(B)** Protopanaxadiol ginsenosides **(C)** Oleanane.

Fortunately, natural products, are one of the most promising options for the management of cancer chemoresistance. It is reported that 49% of anti-cancer drugs which are characterized as multiple targets, high effectiveness, and low toxicity and availability, come directly or indirectly from natural products (Newman and Cragg, 2016). Among these natural products as cancer therapy, none has probably enjoyed as much worldwide prestige and attention as ginseng (Qi et al., 2010). Ginseng is often used in combination with chemotherapeutic drugs to promote chemotherapy. Patients taking ginseng had a 50% lower risk of cancer recurrence compared to patients who do not take ginseng (Yun and Choi, 1995). Shenmai formulation, which contains ginseng, has been proved as a marketed drug in the complementary treatment of cancer chemoresistance, indicating the great treatment potential of ginsenosides when combined with chemotherapeutic agents (Wu et al., 2021; Yang et al., 2020). Ginsenosides, the main active ingredient of ginseng, are usually used in cancer treatment when combined with chemotherapy and this combination provides a better therapeutic intervention than a single usage of ginsenosides or chemotherapy. They are a special group of triterpenoid saponins and composed of 17 carbons and classified into three kinds according to their chemical structure of aglycones: Protopanaxadiol (PPD), protopanaxatriol (PPT) and oleanane (Figure 1). Notably, ginsenoside Rg3 and Rh2, both of which belong to PPD type, are the most intensively researched ginsenosides which possess chemoresistance reversal activity with PPD type. For instance, Li et al. (Li et al., 2016) found that co-treatment of epidermal growth factor receptor-tyrosine kinase inhibitor (EGFR-TKI) and Rg3 may be a promising strategy to delay acquired resistance in clinical treatment. However, to our best knowledge, the chemoresistance-reversal effects of ginsenosides have not been systematically reviewed, which will hamper the usage of ginsenosides in cancer treatment, especially in the development of chemoresistance reversals.

**FIGURE 2:**
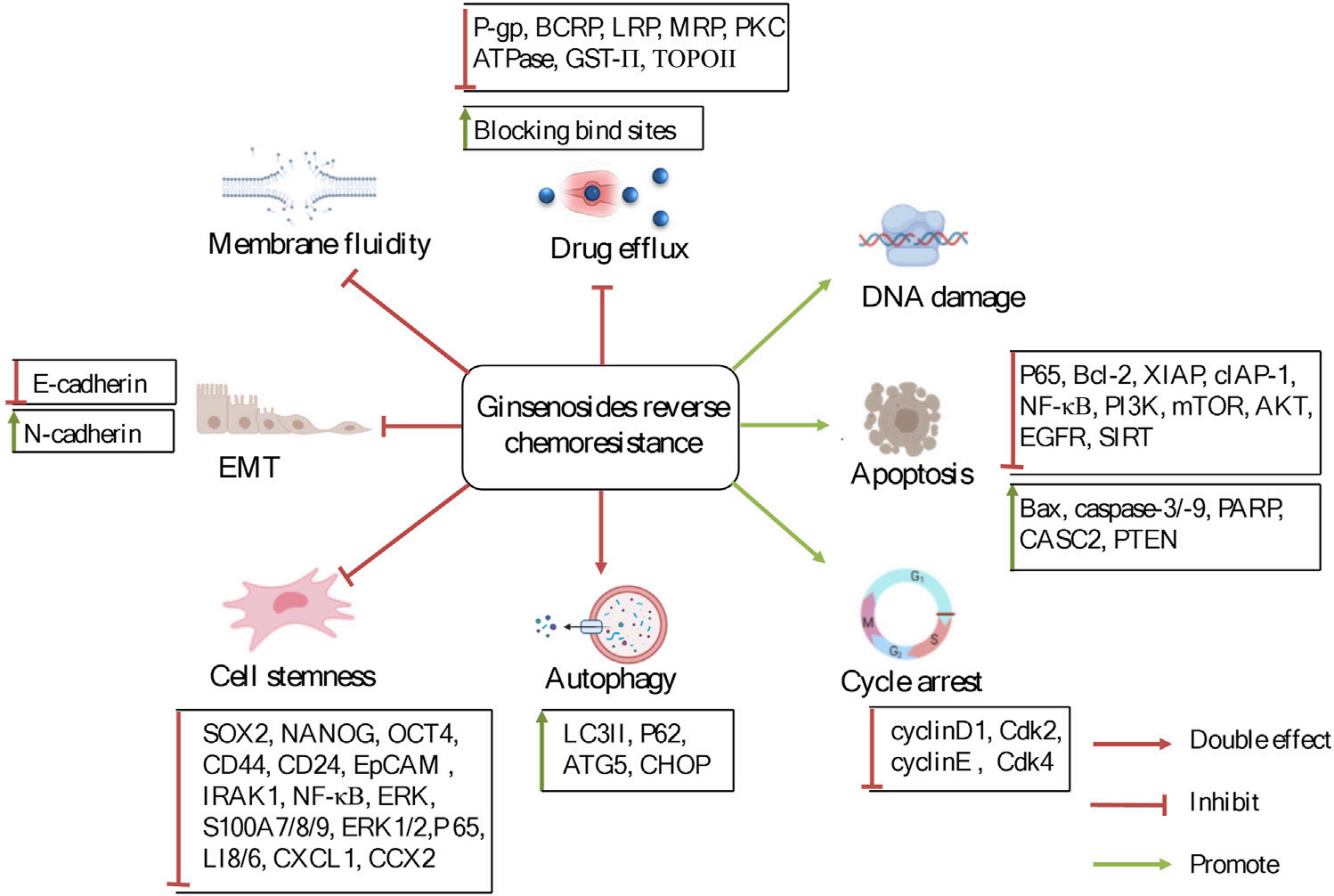
The potentially reversal effects and mechanisms of ginsenosides on chemoresistance in cancer therapy. Ginsenosides were used to treat chemoresistance mainly through the regulation of factors and signaling pathways related to drug transporters, apoptosis, autophagy, and tumor microenvironment.

Here, the medicinal potential and the underlying mechanisms behind the reversal effects of ginsenosides on chemoresistance related to alterations in membrane transporters, apoptosis, autophagy and TME are systematically reviewed and analyzed (Figure 2), and the view that ginsenosides can be used as supplements to reverse chemoresistance is pointed out. We hope this review will lay the foundation for the in-depth investigation of the biochemical mechanisms and pharmacological properties of ginsenosides and benefit the future development and utilization of ginsenosides.

## 2 Inhibiting Membrane Transporters

Overexpression of drug transporters on the plasma membrane has been demonstrated to be a key mechanism conferring chemoresistance (Wu et al., 2008). Generally speaking, these transporters can be classified into ATP-binding cassette (ABC) and non-ABC transporters. Among them, P-glycoprotein (P-gp, gene symbol ABCB1), breast cancer resistance protein (BCRP, gene symbol ABCG2) and multidrug resistance-related protein (MRP, gene symbol ABCC), belong to the ABC transporters family while lung resistance-related protein (LRP) is a non-ABC transporter (Vadlapatla et al., 2013). Additionally, topoisomerase II (TOPO II), glutathione-S-transferase (GST) and protein kinase C (PKC) may also be connected to reverse chemoresistance. However, they usually act as supplements for chemoresistance mediated by drug transporter proteins and genes. Hence, there were few studies to explore their effect alone (Kunze et al., 1991; Daniel, 1993; Beck et al., 1998). Currently, it is recognized that overexpression of these proteins facilitates the efflux of chemotherapeutic agents. The mechanism behind this is that these proteins act as the drug transport barriers between the nucleus and the cytoplasm, inhibit redistribution of drugs as well as prevent chemotherapeutic agents from binding to targets, and in turn directly or indirectly decrease drugs concentration at intracellular targets (Meschini et al., 2002; Thomas and Coley, 2003; Mao and Unadkat, 2015).

Therefore, overexpression and overactivation of these above proteins are considered as the most important reasons resulting in chemoresistance. And an important approach to reverse chemoresistance is to inhibit the expression of drug transporter proteins. And the following mechanisms were involved in the inhibitory effects of ginsenosides on P-gp and non-P-gp transporter proteins.

### 2.1 Inhibiting P-gp-Mediated Chemoresistance

P-gp, which is firstly discovered in Chinese hamster ovarian cancer cells and is closely related to drug-resistant cells, is mainly located in the cell membrane, and account for a low proportion in the endoplasmic reticulum, mitochondrial, and Golgi body (Juliano and Ling, 1976; Szabó et al., 2000). But it is worth noting that P-gp in the mitochondria pumps the drug from the cytoplasm into the mitochondria, whereas P-gp on the cell membrane pumps the drug out of the cells (Munteanu et al., 2006). Therefore, overexpression of P-gp in different locations may result in drug redistribution and accumulation at targets. Moreover, as a classic mechanism of chemoresistance, the activation of P-gp is associated with chemoresistance to several types of chemotherapeutic agents, such as anthracycline, vincristine and paclitaxel (Gottesman et al., 2002; Bikadi et al., 2011). At present, P-gp mediated chemoresistance can be reversed by three generation inhibitors (Chen Z et al., 2016). Although these inhibitors exert an inhibitory effect on P-gp, their adverse effects limit their implication, such as low specificity and affinity, undesirable drug-drug interactions and toxicity to normal cells in clinical trials (Daenen et al., 2004; Kelly et al., 2011; Syed and Coumar, 2016). Therefore, it is of great necessity to seek out novel P-gp inhibitors that are not only highly effective and low toxic but also obviously potentially reversal agents of P-gp mediated chemoresistance.

Ginsenosides can efficiently reverse chemoresistance as P-gp inhibitors (Chen et al., 2014). Compared to classic P-gp inhibitors, ginsenosides appear to be multi-targeted P-gp inhibitors, whose mechanisms include downregulating the expression of P-gp, competitively or non-competitively blocking the binding site of P-gp and suppressing the cell membrane fluidity.

#### 2.1.1 Downregulating Expression of P-gp

The pharmacological mechanism of chemoresistance is mainly attributed to a decreased accumulation of intracellular drugs mediated by P-gp overexpression. Thereby, inhibiting overexpression of P-gp and MDR1 can result in reversal of chemoresistance (Fu et al., 2013).

Studies have found that ginsenosides in combination with chemotherapeutic agents exert a remarkably inhibitory effect on chemoresistance through inhibiting overexpression of P-gp and MDR1 and consequently suppressing drug effluxes. For example, Fu et al. found that ginsenosides might facilitate Eca109/DDP cells more sensitive to cisplatin (DDP). And their study showed that co-treatment of ginsenoside Rg3 and DDP decreased expression of P-gp more than treatment of DDP alone, suggesting the decreased expression of P-gp might be responsible for ginsenoside Rg3-mediated reversal of chemoresistance. Similarly, Ma and his coworkers (Ma et al., 2019) demonstrated that ginsenoside Rh2 could be a potential agent to reverse chemoresistance when co-treated with oxaliplatin in LoVo/L-OHP cells through decreasing the protein and mRNA levels of P-gp. Also, another study by Li and Chen (Li and Chen, 2013) indicated that ginsenoside Rh2 significantly decreased adriamycin (ADM/Adr) or fluorouracil (FU) efflux in MCF-7/ADM cells. Further, Rh2 could reverse chemo-resistant cells to chemotherapeutic agents by depressing the expression of P-gp. Additionally, ginsenosides can inhibit overexpression of P-gp especially in the nuclei and mitochondria, and thereby change drug distribution. Zhang et al. (Zhang et al., 2012c) showed that treatment of 20 (S)-Rh2 not only decreased IC_50_ of MCF-7/Adr cells by inhibiting expression of P-gp but also promoted the redistribution of ADM. With the suppressive effect on expressions of P-gp, 20 (S)-Rh2 could up-regulate the rate and amount of ADM entering intracellular sites in MCF-7/Adr cells, especially in the nuclei and mitochondria where P-gp was over-expressed, which ultimately contributed to reverse chemoresistance and enhance cell apoptosis induced by ADM.

Further studies also revealed the underlying mechanism which contributed to the regulation of ginsenosides on overexpression of P-gp. Ginsenosides could also reverse chemoresistance through inhibiting the expression of MDR1 gene and increasing the ubiquitination of MDR1. MDR1 stability is dependent on ubiquitin-dependent protein degradation (Zhang et al., 2004). A study (Pokharel et al., 2010) firstly found that ginsenoside Rd, Re, Rb1, and Rg1, especially Rd, decreased MDR1 protein levels in MCF-7/ADR cells, which could be reversed by MG-132, a proteasome inhibitor, suggesting that the proteasomal degradation pathway was responsible for turnover of Rd-mediated MDR1. However, further study showed that Rd did not change mRNA or nuclear protein levels of key transcription factors including hypoxia-inducible factor-1 *α* (HIF-1 *α*), C/-enhancer-binding protein *β* (C/EBP *β*), forkhead box-containing protein, O subfamily1(FoxO1), or Y-box binding protein-1(YB-1), which regulated human MDR1 gene transcription or affected the pregnane X receptor (PXR) dependent transcription activity, showing that the effect of Rd on chemoresistance was not related to the transcription of MDR1 gene. Additionally, Rd increased ubiquitination of MDR1 and consequently reversed doxorubicin (DOX)-resistance in MCF-7/Adr cells, which increased MDR1 protein levels. Further study by Zhang et al. (Zhang et al., 2012a) aimed to elucidate the key factors that decreased ABCB1 expression, and altered cellular pharmacokinetics of Adr, revealing the synergistic mechanism of 20 (S)-Rh2 on MCF-7/Adr cells when co-treated with Adr. The mitogen-activated protein kinase (MAPK)/nuclear factor (NF)-*κ*B pathway, translocation and binding activity of NF-*κ*B and the binding capability of NF-*κ*B to the human MDR1 promoter were inhibited by 20 (S)-Rh2 in MCF-7/Adr cells. Moreover, inhibiting the MAPK/NF-*κ*B pathway brought about a decrease in the expression of ABCB1 and the cellular pharmacokinetics regulated by Adr was also significantly altered by inhibiting NF-*κ*B. Overall, 20 (S)-Rh2 might down-regulate Adr-induced overexpression of ABCB1 in MCF-7/Adr cells by alleviating the MAPK/NF-*κ*B pathway.

#### 2.1.2 Blocking the Binding Site of Chemotherapeutic Agents

In addition, blocking the binding site of chemotherapeutic drugs and P-gp is efficient to reverse chemoresistance. There are two types of blockers, including competitive and non-competitive ones. The competitive blockers have high affinity and refer to the ones that leave little place for the binding of P-gp to other chemotherapeutic agents, which result in decreased activity of P-gp (Srivalli and Lakshmi., 2012). In contrast, the non-competitive blockers, substrates of P-gp, are highly effective, reversible, and non-competitive inhibitors of P-gp (Dewanjee et al., 2017).

At present, there were lots of studies showing that ginsenosides could decrease drug effluxes through competitively or non-competitively inhibiting drugs from being bound with P-gp. On one hand, ginsenosides always inhibit chemotherapeutic agents from being bound to these proteins by competitively blocking their binding site and therefore resisting drug efflux, without any effects on the mRNA and protein expressions of drug-related proteins. For example, Choi et al. (Choi et al., 2003) found that protropanaxatriol ginsenosides (PTG) including ginsenoside Rg1 and Re had the potential to reverse MDR by competitively blocking the [^3^H]-azidopine binding site in DOX resistant AML-2/DX100 cells. Similarly, Gao et al. (Gao et al., 2002) found that Rg3 improved the sensitivity of K562/ADM cells to ADM but failed to affect the growth of K562/ADM cells expressing P-170. Thus, it was speculated that 20 (R)-ginsenoside Rg3 could reverse P-gp-mediated MDR through direct interaction with P-gp in breast cancer treatment. Kim et al. (Kim et al., 2003) revealed that IC_50_ values of vincristine (VCR), DOX, colchicine (COL) and VP-16 in KBV20C were decreased and the intracellular accumulation of drugs was increased in KBV20C cells following Rg3 treatment. Without any effect on MDR1 gene expression or P-gp level, Rg3 resisted vinblastine efflux and reversed chemoresistance to those drugs in KBV20C cells, properly owing to its competition with [^3^H] azidopine for binding to the P-gp. It was suggested Rg3 could specifically inhibit P-gp-mediated drug accumulation. Thus, reversal of chemoresistance mediated by Rg3 was redounded to its competition with anticancer drugs for binding to P-gp thereby blocking drug efflux. In addition, Rb1, a major component of ginseng, exerted a synergistic effect with vincristine on chemoresistance in HL60/VCR cells but failed to inhibit the expression of MDR1 (Li et al., 2005). It was worth noting that a membrane breaker was used for the first time in this study to explore the mechanism of why P-gp activity was decreased by Rb1. Although P-gp on the cell membrane was decreased significantly in the presence of Rb1 before cell membrane integrity was broken, the total P-gp was unaffected, indicating that Rb1 has no effect on P-gp expression but P-gp function. Hence, the P-gp inhibitory effect of Rb1 was possibly dependent on the calcium channel since Rb1 was a blocker of the calcium channel, similar to Vp. Consistently, Chen et al. (Chen G et al., 2016) explored the reason why PPD 12, a derivative of 20(S)-protopanaxadiol (PPD), enhanced the sensitivity of MDR cells to ADM. This new compound strengthened the accumulation of ADM and Rho123 *via* blocking the efflux. As a substrate of P-gp, PPD12 inhibited the transport function of ABCB1 by stimulating the ATPase activity but failed to alter mRNA or protein expression, indicating that PPD is a substrate of P-gp as the first generation of P-gp inhibitor. Moreover, a predicted binding mode showed hydrophobic interactions of PPD12 within the large drug-binding cavity of ABCB1. Residues in the drug-binding pocket of ABCB1 were bound with P-gp stably through hydrogen bonding and hydrophobic interactions. Notably, PPD12 in non-toxic concentrations sensitized chemo-resistant cells to their anticancer substrates better than either the PPD. Additionally, co-treatment of PPD12 and ADM treated in the KB/VCR xenograft mice model inhibited tumor growth without decreasing weight. Similarly, Rg5 could reverse chemoresistance through inhibiting the function of ABCB1 by stimulating the ATPase activity both in A2780/T cells and A549/T cells (Feng et al., 2020). The underlying mechanism was partially attributive to its upregulation on the activity of ABCB1 ATPase. As Rg5 activated the ATPase of ABCB1, it might be a substrate of ABCB1. However, it inhibited Vp stimulated ATPase activity, which showed that Rg5 was bound to P-gp with high affinity and prevented other agents from being bound to the transporters, resulting in decreased activity of ABCB1 transporter. On the other hand, ginsenosides exerted an inhibitory effect on chemoresistant cells by non-competitively blocking the activity of P-gp. For instance, 20 (S)-Rh2 was a non-competitive P-gp inhibitor (Zhang et al., 2010). It significantly inhibited the efflux rates of three typical P-gp inhibitors, involving digoxin, fexofenadine and etoposide and the inhibitory action of 20 (S)-Rh2 on the function of P-gp could persist for 3 h at least after washed in MCF-7/Adr cells. Unlike P-gp substrates, 20 (S)-Rh2 reduced the Vp-stimulated P-gp ATPase activity close to the basal level and UIC2 binding fluorescence. Furthermore, 20 (S)-Rh2 promoted absorption and bioavailability of etoposide significantly without affecting terminal elimination half-time and P-gp expression *in situ* and *in vivo*. Collectively, 20 (S)-Rh2 exerted an inhibitory effect in a noncompetitive instead of a competitive manner. Similarly, Zhao et al. (Zhao et al., 2009) held that aPPD, the final deglycosylation metabolite of the PPD group in the gastrointestinal tract, was exactly a non-competitive and reversible inhibitor of P-gp with high efficiency. This study revealed that IC_50_ values of aPPD-induced cytotoxicity in drug sensitive P388wt and drug resistant P388adr cells were similar, suggesting that aPPD is impossible to be a substrate of P-gp. In addition, the level of calcein accumulation was enhanced and a similar inhibitory effect on the activity of P-gp was observed in MCF-7/Adr and P388/Adr cells in the presence of aPPD. Differently from 20 (S)-Rh2, the activity of P-gp immediately returned to the control levels after removal of aPPD and aPPD exerted no effect on ATPase. Another study (Liu et al., 2004) also indicated that the blockage of P-gp activity was highly reversible after aPPD was washed out, which suggested recovery of P-gp activity. Furthermore, aPPD did not affect ATPase activity of P-gp. Also, the activity of aPPD on chemoresistance was observed *in vivo*. Paradoxically, although both aPPD and 20 (S)-Rh2 were not substrates of P-gp, the recovery time of P-gp activity was different when aPPD and 20 (S)-Rh2 was removed due to their different chemical structures.

#### 2.1.3 Suppressing the Lipid Fluidity and Modulating Lipid Rafts

The fluidity of the membrane refers to the extent of the molecular disorder and molecular motion within a lipid bilayer. Cholesterol, the main ingredient of a lipid bilayer, is responsible for lipid fluidity owing to its function and activity (Yeagle, 1991). Moreover, cholesterol is a key factor to maintain the complete structure and function of lipid rafts. Depletion of cholesterol leads to rupture of lipid rafts and confusion of cell function (Li et al., 2006). Lipid rafts are detergent-resistant microdomains of the plasma membrane with low-density and enriched in cholesterol and glycosphingolipids. Structurally, quantities of drug transporters, such as P-gp, are located in the cell membrane and lipid rafts. Therefore, lipid fluidity and lipid rafts play a central role in chemoresistance in cancers though affecting the function of drug transporters.

Ginsenosides-induced reversal of chemoresistance mediated by P-gp has been achieved through alteration of lipid fluidity and modulation of lipid rafts. For instance, a study performed by Kwon et al. (Kwon et al., 2008) showed that because of the structural similarity of Rg3 and cholesterol, Rg3 decreased the membrane fluidity and in turn blocked drug efflux induced by P-gp in drug resistant KB V20C cells but not in drug sensitive KB V20 cells, which resulted in reversal of P-gp-mediated chemoresistance. Moreover, Rg3 induced a significant increase of fluorescence anisotropy in KB V20C cells, suggesting that Rg3 significantly decreased cellular lipid membrane fluidity in KB V20C cells, thereby decreasing drug effluxes. Notably, the inhibitory effect could be reversed when Rg3 was removed. Also, the longevity of mice implanted with Adr-resistant P388 leukemia cells was remarkably promoted by Rg3. Collectively, Rg3 exerted an inhibitory effect on the membrane fluidity of drug-resistant cells, consequently reversing chemoresistance. Also, ginsenoside Rp1, a novel semi-synthesized agent from Rg5 and Rk1, had an inhibitory effect on lipid fluidity (Yun et al., 2013) By competing with cholesterol for incorporation into lipid rafts, ginsenoside Rp1 induced lipid raft clustering and accordingly decreased membrane fluidity in drug resistant NCI/ADR-RES and DXR cells, which caused translocation of MDR-1 out of lipid rafts, inhibited the activity of MDR1 and augmented an accumulation of DOX or rho 123. Moreover, the inactivation of Src, a kinase located in lipid rafts, led to a decrease of MDR1 in DXR cells treated by co-treatment of Rp-1 and actinomycin D(ActD). Overall, as a cholesterol depletor, Rp1 reversed chemoresistance *via* modulating lipid rafts and inactivating Src and MDR1.

### 2.2 Downregulating Expression of Non-P-gp Proteins

Three transporters, involving MRP, BCRP, and LRP, belong to non-P-gp proteins. MRP functions as the major exporter of organic anions and overexpression of MRP can confer resistance to platinum-containing chemotherapeutic drugs, such as DDP (Surowiak et al., 2006) BCRP, a semi-transport protein, delivers chemotherapeutic drugs through forming homodimers or oligomers and consequently develops transmembrane channels. Overexpression of BCRP is reported to confer chemoresistance for a wide range of chemotherapeutics such as MX, camptothecin derivates, flavopiridol, and methotrexate (Ross et al., 1999). LRP mediates resistance of certain drugs that cannot be mediated by P-gp and MRP, such as carboplatin and alkylating agents, all of which target DNA (Meschini et al., 2002). Collectively, overexpression of one or multiple proteins mentioned above may lead to chemoresistance. Interestingly, the suppression of these proteins and P-gp are observed simultaneously.

Ginsenosides Rg3 and Rh2 are the most studied saponins that can inhibit the expression of transporters. Zhang et al. (Zhang et al., 2002) found that Rg3 might act as a promising candidate for chemoresistance in human lung adenocarcinoma (A549/DDP cells) time–dependently through inhibiting expression of chemoresistance-related proteins, including MDR1, MRP and LRP. Co-treatment of DDP and Rg3 decreased not only IC_50_ values of DDP in A549/DDP cells but also the mRNA and protein levels of MDR1, MRP and LRP. Liu et al. (Liu Cet al., 2018) obtained similar results from the previous findings. Moreover, the nude mice bearing human lung adenocarcinoma that received co-treatment of DDP and Rg3 had obviously reduced tumor volumes and weights compared with animals treated with DDP alone. Moreover, Rg3 treatment enhanced uptake of technetium-99m labeled hexakis-2-methoxyisobutylisonitrile (99mTc-MIBI) suggesting that Rg3 can sensitize chemotherapy *in vivo*. Consistently, another study (Liu GW et al., 2018) showed that ginsenoside Rh2 reversed resistance of chemotherapeutic agents through inhibiting multiple drug resistance related proteins in HCT-8/5-FU and LoVo/5-FU cells. Expressions of MRP1, MDR1, LRP and GST were obviously inhibited in the presence of Rh2.

Furthermore, the metabolites of ginsenosides exert inhibitory effects on MDR tumor cells synergistically as well. Jin et al. (Jin et al., 2006) suggested that PPD is a useful ingredient to improve chemotherapy responsiveness as a novel BCRP inhibitor. PPD reinforced the antiproliferative action of MX on human breast carcinoma MCF-7/MX cells that overexpressed BCRP. And MX retention was lower in MCF-7/MX cells than MCF-7 cells, suggesting that the extruding MX ability in MCF-7/Adr cells might be related to BCRP. Additionally, uptake of MX was obviously enhanced and the activity of BCRP ATPase was inhibited in the presence of PPD in MCF-7/MX cells. Therefore, it was indicated that PPD significantly reverses the resistance of MCF-7/MX cells to MX as a BCRP inhibitor in a synergistic fashion. In conclusion, overexpression of chemoresistance-related proteins (P-gp, MRP, LRP, BCRP and GST) could be reversed by co-treatment of chemotherapeutic agents and ginsenosides.

## 3 Regulating Apoptosis and Autophagy

Apoptosis and autophagy are two different forms of programmed cell death, and there may be a functional relationship between them. Presently, studies have found that there are roughly three relationships between autophagy and apoptosis. 1) Autophagy usually precedes apoptosis and then activates apoptosis(Cui et al., 2007). 2) In some cases, such as mitochondrial damage caused by stimulation by oxidation, ischemia/reperfusion and toxic compounds, etc. (Kanzawa et al., 2003), autophagy can protect cells from apoptosis and necrosis. 3) Autophagy and apoptosis promote cell death together (Yanagisawa et al., 2003). In short, autophagy not only has been suggested as a possible mechanism for non-apoptotic death but also represents a survival strategy. However, the specific transformation mechanism between the two types of cell death is not remains unclear. To our best knowledge, ginsenosides reversed chemoresistance by modulating both of apoptosis and autophagy. Therefore, the chemoresistance-reversal mechanism involved in ginsenosides induced apoptosis and autophagy were introduced together as follow.

### 3.1 Inducing Cancer Cell Apoptosis

Apoptosis, known as programmed death, is one of the important life phenomena in the biological world. Generally, the Fas pathway, mitochondrial passage, adenylyl-cyclase pathway and p53 activation pathway mediate apoptosis. They target Bax and suppress anti-apoptotic proteins such as Bcl-2, finally triggering apoptosis (Lee et al., 2020). Data has suggested that inducing apoptosis sensitized cancer cells to chemotherapeutic drugs, such as 5-FU, DOX and ActD (Boyer et al., 2004; Fulda and Debatin, 2004). Therefore, inducing apoptosis can contribute to the suppression of chemoresistance (Schmitt and Lowe, 2001).

Ginsenosides act as apoptosis inducers to reverse chemoresistance. Investigations had indicated that ginsenosides induced apoptosis in different cancer cell lines, resulting in the reversal of chemoresistance *in vitro* and *in vivo*. For instance, Hu et al. (Hu et al., 2010) revealed that the chemoresistance reversal effect of Rh2 in A549/DDP cells could be achieved through the mitochondrial apoptosis pathway. Intracellular calcium ion concentration was higher after DDP treatment than after co-treatment of DDP and Rh2. After co-treatment of Rh2 and DDP, the expression of cyto C in the cytoplasm was higher than that in mitochondria. Moreover, caspase 3 was motivated and highly expressed in A549/DDP cells. In contrast, the expression of cyto C was higher in the mitochondria than that in the cytoplasm and the nucleus was reduced after treatment of Rh2 or DDP alone. Therefore, the combination of Rh2 and DDP significantly increased cell apoptosis in the drug resistant cancer cells. Consistently, Ma et al. (Ma et al., 2019) showed that the reversal effect of Rh2 on oxaliplatin-resistant colon cancer cells was correlated with apoptosis activation as well. Rh2 could reverse L-OHP induced resistance in LoVo/L-OHP cells through inhibiting proliferation and protein and mRNA levels of Bax and promoting protein and mRNA levels of Bax and caspase-3.

More importantly, further in-depth studies showed that inducing apoptosis can be mediated by different signaling pathways which could be clarified as follows.

#### 3.1.1 Modulating the CASC2/PTEN/Akt Signaling Pathway

LncRNA cancer susceptibility candidate 2 (CASC2) and protein tyrosine phosphatase gene (PTEN) have been characterized as tumor suppressors and efficient suppressive regulators of cancers. Regulation of CASC2 and PTEN induced by AKT is closely associated with chemoresistance of tumors. For example, Feng et al. (Feng et al., 2017) found that inhibition of the CASC2/PTEN/Akt pathway sensitized DDP-resistant cervical cancer cells to DDP since overexpression of CASC2 reduced IC_50_ values in HeLa/DDP and CaSki/DDP cells, while CASC2 knockdown promoted IC_50_ values. Furthermore, CASC2 inhibits miR-21 and p-AKT protein level and promotes PTEN protein level, indicating that CASC2 might sensitize cancer cells to drugs through regulating PTEN and Akt pathways. Similarly, PTEN has also been regarded as a therapeutic target for reversing chemoresistance (Xia et al., 2013). Therefore, suppression of the CASC2/PTEN/Akt pathway plays an important role in the reversal of chemoresistance.

Co-treatment of Rg3 and gemcitabine (GEM) can inhibit the growth of drug-resistant cells through the CASC2/PTEN/Akt pathway. Zou et al. (Zou et al., 2020) found that inhibition of the PTEN/Akt signaling was involved in the reversal effect of Rg3 in GEM-resistant pancreatic cancer. The cell growth and colony formation of Panc-1/GEM and SW1990/GEM cells were suppressed after treatment of GEM and Rg3. At the same time, Rg3 could activate the CASC2/PTEN signaling, inhibit cell growth and induce cells apoptosis concentration-dependently in Panc-1/GEM cells and SW1990/GEM cells. Moreover, the expression of PTEN was positively related to CASC2, suggesting that Rg3 modulates PTEN signaling through activating CASC2 expression. Additionally, a significant decrease in tumor volume and weight were observed in the nude mice implanted with GEM-resistant pancreatic cancer cells when treated by Rg3. Overall, Rg3 increased cell apoptosis and consequently reversed chemoresistance through modulating the CASC2/PTEN signaling pathway in GEM-resistant pancreatic cancer.

#### 3.1.2 Modulating the AKT/SIRT1 Pathway

Sirtuin1 (SIRT1) can deacetylate various histone and non-histone substrates, such as p53, c-MYC and FOXO, therefore altering DNA repair, cell cycle, apoptosis and development of chemoresistance (Chen et al., 2011; Wang et al., 2013; Mao et al., 2014). Also, SIRT1 inactivates the AKT pathway in a SIRT1 deacetylase-dependent manner (Wang et al., 2015). Therefore, regulation of the SIRT1/AKT pathway could be a possible strategy for reversing chemoresistance.

There was a study confirming that the combination of ginsenoside Rp1 and ActD exerted a synergistic sensitization on the drug resistant cancer lines by modulating the AKT/SIRT1 pathway (Yun et al., 2020). Treatment of ActD or Rp1 alone mildly inhibited the cell growth while the inhibitory effect of co-administration of Rp1 and ActD was significantly promoted in drug-resistant LS513 colon cancer. Importantly, Rp1 re-sensitized the drug resistant cells to ActD through increasing apoptosis and aggravating DNA damage. Further researches suggested that Rp1 could attenuate the up-regulation of SIRT1 caused by ActD and consequently increase apoptosis through enhancing p53 acetylation in drug resistant cells. Meanwhile, co-treatment of Rp1 and ActD also decreased AKT activation to downregulate the expression of SIRT1, and ultimately activated cells apoptosis as well as reversed chemoresistance in tumor cells. More, tumor growth was significantly inhibited when ActD combined with Rp1 in nude mice implanted with LS513 cells as well. Taken together, modulation of the AKT/SIRT1 pathway was involved in Rp1-reversed drug resistance.

#### 3.1.3 Modulating the EGFR/PI3K/AKT Signaling Pathway

Epidermal growth factor receptor (EGFR), known as a key factor in the development of tumor, can bind with a ligand, which leads to autophosphorylation of the receptor, activate downstream signal transduction and activity, involving Ras/MAPK, PI3K/AKT, STAT, and Src family kinases, and promote proliferation, survival, invasion, and migration of tumor cells (Wieduwilt and Moasser, 2008). Among them, the EGFR/PI3K/AKT pathway indirectly regulates tumor cell apoptosis through maintaining the balance between cell proliferation and apoptosis (Hong and Fan, 2019). Therefore, inhibition of the EGFR/PI3K/AKT pathway could be a viable strategy for overcoming chemoresistance through affecting apoptosis.

Rg3-mediated inactivation of the EGFR/PI3K/AKT pathway is an efficient method to induce cancer cell apoptosis, which has been proved by Jiang and his coworkers (Jiang et al., 2017). The sensitivity of erlotinib to pancreatic cancer cells was significantly enhanced when co-treatment with Rg3 *in vitro* and *in vivo*. The cell viability, growth and formation of cell colonies in BxPC-3 and AsPC-1 cells were inhibited when exposed to different concentrations of Rg3 and erlotinib. Likewise, apoptosis induced by erlotinib and cleavage of caspase-3, caspase-9 and PARP were obviously promoted by Rg3 in BxPC-3 and AsPC-1 cells. Furthermore, lower expression levels of p-EGFR, p-PI3K and p-AKT were observed when combined with Rg3, which revealed that Rg3 downregulates expressions of p-EGFR, p-PI3K, and p-AKT, thereby sensitizing pancreatic cancer cells to erlotinib. Similarly, the study *in vivo* showed Rg3 inactivated the EGFR/PI3K/AKT signal pathway. Moreover, the tumor volume was decreased by co-treatment of erlotinib and Rg3 in nude mice implanted with BxPC-3 cells. In conclusion, downregulating the EGFR/PI3K/AKT signal pathway, which induced cell apoptosis, was a vital mechanism related to Rg3-mediated reversal of chemoresistance.

#### 3.1.4 Modulating the PI3K/AKT/mTOR Pathway

Activation of the PI3K/AKT/mTOR pathway is a common feature of a wide range of human cancers (Brown et al., 2008). Upregulation of the PI3K/AKT/mTOR pathway contributes to cancer cell survival, acquires chemoresistance, which was related to inhibition of apoptosis and cell cycle progression, differentiation and growth (Choo and Blenis, 2006). Thus, it is believable that activating the PI3K/AKT/mTOR signaling can effectively result in chemoresistance reversal.

Ginsenoside Rg1 could induce apoptosis in paclitaxel-resistant nasopharyngeal cancer cells which might be correlated to suppression of the mTOR/PI3K/AKT pathway (Li et al., 2019). Rg1 increased Bax and decreased Bcl-2 expression and induced cycle arrest in the S phase in SUNE1 cells. Additionally, Rg1 decreased the phosphorylation of mTOR, PI3K in a concentration dependent manner without apparent effects on the total PI3K, AKT and mTOR, suggesting that Rg1 inactivated the TOR/PI3K/AKT pathway in SUNE1 cells. Taken together, Rg1 is considered as a supplementary anticancer agent to reverse chemoresistance due to its inactivation of the TOR/PI3K/AKT pathway in paclitaxel-resistant nasopharyngeal carcinoma.

#### 3.1.5 Modulating the NF-*κ*B Pathway

Constitutive activation of nuclear factor-*κ*B (NF-*κ*B) in cancer treatment induces tumor promotion, angiogenesis, metastasis, apoptosis, and resistance to anticancer drugs (Garg and Aggarwal, 2002; Pikarsky et al., 2004). Therefore, inhibition of NF-*κ*B is an efficient approach to increase apoptosis and reverse chemoresistance.

Growing evidence implied that the combination of ginsenosides and chemotherapeutic agents exerted an inhibitory effect *via* inactivation of the NF-*κ*B pathway in cancer cells. For instance, ginsenoside Rg3 combined with the conventional drugs, involving docetaxel (TXT), paclitaxel, DDP and Adr, resisted the growth of HCT116 and SW620 colon cancer cells and induced apoptosis *via* inhibiting NF-*κ*B (Kim et al., 2009). Co-treatment of Rg3 and TXT obviously induced apoptosis through regulating apoptotic related genes, including inhibiting the expression level of Bcl-2, XIAP and cIAP-1 and promoting the expression of Bax, caspase-3 and caspase-9. In agreement with this, Rg3 resisted the NF-*κ*B DNA binding activity, TNF-*α*-induced transcriptional activation of NF-*κ*B and AP-1 activity and notably promoted apoptosis and sensitivity of chemotherapeutic drugs in colon cancer cells. Similarly, Kim et al. (Kim et al., 2010) found that higher doses of Rg3 could enhance the sensitivity of prostate cancer to TXT through downregulation of the NF-*κ*B pathway. Inhibition of NF-*κ*B pathway and cells growth and induction of apoptosis was observed after Rg3 treatment in prostate cells (LNCaP, PC-3, and DU145), especially LNCaP cells. Importantly, co-treatment of Rg3 and TXT caused a significant reduction in the expression of G0/G1 regulators cyclin D1, cyclin E, Cdk2, and Cdk4. Moreover, another study by Yuan et al. (Yuan et al., 2017) found that the synergistic inhibitory effect of the combination of Rg3 and paclitaxel in triple-negative breast cancer (TNBC) cells, including three cancer cell lines MDA-MB-231, MDA-MB-453, and BT-549, resulted from regulation of apoptotic proteins and genes and inactivation of NF-*κ*B. Additionally, reduction of tumor volumes and weights in the nude mice implanted with TNBC cells *in vivo* after co-treatment of Rg3 and paclitaxel was observed. Therefore, the treatment of ginsenoside Rg3 could serve as a novel strategy in treating a line of cancers with a high incidence and mortality *via* inhibiting the NF-*κ*B pathway.

### 3.2 Modulating Cell Autophagy

Autophagy is a lysosome-mediated protein and organelle degradation process that is characterized by the formation of autophagosomes (Mizushima and Klionsky, 2007). Intensive studies have suggested that autophagy can serve as both an inhibitor and a motivator of cancer cells relying on the degree of induction (autophagic flux) and stage of tumor progression (Eskelinen, 2011). Similarly, the chemo-resistant mechanism associated with autophagy is separated into two aspects. Some cases maintain high basal autophagic flux, resulting in intrinsic resistance to chemotherapeutic drugs while others gradually acquire chemoresistance by increasing autophagic fluxes in response to the treatment of chemotherapeutic drugs. Paradoxically, an increase in autophagic flux may also induce tumor cell death in some cases, leading to remarked inconsistency across studies (Sheng et al., 2018). In brief, a better understanding of the dual role of autophagy in cancer is necessary to figure out the acquirement of chemoresistance.

Ginsenosides can activate autophagy to reverse chemoresistance. For example, Rp1 was efficient to resist proliferation in paclitaxel-resistant nasopharyngeal cancer by promoting autophagic cell death (Li et al., 2019). The data showed that Rg1 increased the protein levels of LC3-II and decreased the expression of p62 in SUNE1 cells. Taken together, the activation of autophagy was correlated with the reversal of chemoresistance mediated by Rg1.

In contrast, autophagy inhibition is critical for ginsenosides to reverse chemoresistance. Wang et al. (Wang et al., 2019) found that 20(S)-Rg3 sensitized PC-9-resistant (PC-9R) and HCC827-resistant (HCC827R) cells to icotinib through suppressing the late maturation or degradation stage of autophagy. The levels of LC3II protein, p62 protein and the number of LC3II puncta in PC-9 and HCC827 cells were increased by Rg3. However, protein levels of LC3II and p62 were not increased anymore when Rg3 combined with CQ, which indicated that the increased LC3 puncta formation accompanied by increased p62 levels in Rg3-treated cells may attribute to suppression of the late maturation or degradation stage of autophagy. Consistently, their results showed that inhibition of autophagy by Rg3 could reverse chemoresistance in PC-9R xenograft models. Another study performed by Kim et al. (Kim et al., 2014) showed that autophagy was often regarded as a cell survival mechanism in cancer cells exposed to DOX while 20 (S)-Rg3 sensitized hepatocellular carcinoma to DOX through inhibiting autophagy, which resulted in cell death. More importantly, CHOP as well as the ATG-5 dependent autophagic pathway was involved in Rg3-inhibited autophagy. Rg3 did not increase LC3II and p62 protein levels anymore when ATG5 gene was abolished. Moreover, knockdown of CHOP repressed 20 (S)-Rg3-induced death of HepG2 cells while upregulation of CHOP could promote TRAIL-induced apoptosis. Additionally, a decrease in tumor weight and volume was observed after treatment of Rg3 *in vivo*. Collectively, 20 (S)-ginsenoside Rg3 was a novel inhibitor of autophagy through increasing LC3II and p62 protein levels in a CHOP and ATG-5 dependent autophagic manner and thus sensitized hepatocellular carcinoma to doxorubicin.

## 4 Modulating Tumor Microenvironment (TME)

Tumor microenvironment (TME), including smooth muscle cells, endothelial cells, fibroblasts of various phenotypes, myofibroblasts, mast cells, T cells, B cells, natural killer, neutrophils, granulocyte, and antigen-presenting cells is a complex and constantly changing matrix surrounding tumor cells. The interaction of these components in TME has affected the biological behavior of tumors including epithelial-mesenchymal transition (EMT), characteristics maintenance of cancer stem cells (CSCs), invasion, metastasis, and chemoresistance (Polyak et al., 2009; Hale et al., 2013). CSCs, typical cells in TME, show a high degree of uncertainty and instability. They regulate the expression of transporters and apoptotic genes, the activity of microsomal multifunctional oxidases, DNA damage repair, and activation of key cell signaling pathways, which have become one of the main theories that produce chemoresistance. EMT is a phenomenon produced by epithelial cells under the action of matrix metalloproteinases, adhesion molecules, selectins and other factors. It is one of the initial development methods of CSCs and has the potential to endow CSCs with chemoresistance, viability and metastatic ability (Huang et al., 2012). Additionally, hypoxia is the most prominent sign of TME and results from tumor cell proliferation that is faster than angiogenesis or abnormal new blood vessels (Challapalli et al., 2017). Studies have revealed that hypoxia can promote EMT and cancer cell stemness, which facilitates cell survival and acquires chemoresistance (Cosse and Michiels, 2008; Bharti et al., 2016). Therefore, it is important to modulate the resistance of solid tumors to chemotherapeutic agents by modulating TME.

Studies showed that ginsenosides suppressed the ovarian stem cancer cells through inhibiting the process of EMT. For example, Kala et al. (Kala, 2014) found that Rb1 or CK combined with DDP or paclitaxel significantly reversed chemoresistance in a dose and time dependent manner through inhibiting ovarian stem cancer cells. Co-treatment of ginsenosides and chemotherapeutic agents obviously inhibited tumor sphere forming ability and cell viability through downregulating E-cadherin and up-regulating N-cadherin in HEYA8 and SKOV-3 cells. Therefore, modulating EMT to inhibit the cell stemness was the reason why Rb1 or CK enhanced the sensitivity of cancer cells to chemotherapeutic agents. In another case, Wang et al. (Wang et al., 2018) found co-treatment of Rg3 and DDP exerted an important role in hypoxic lung cancer therapy. Rg3 could sensitize hypoxia human NSCLC cells to DDP by inhibiting the NF-*κ*B pathway thereby retaining the progress of EMT and stemness evidenced as reduced expressions of E-cadherin, N-cadherin, sex determining region Y-box 2 (SOX2), NANOG, OCT4, and CD44. Moreover, consistent results both *in vivo* and *in vitro* were observed. Collectively, Rg3 could sensitize hypoxic lung cancer cells to DDP through preventing NF-*κ*B induced EMT and stemness. Also, Rg3 (Tan et al., 2020) could target CSCs and reverse osimertinib resistance in H1975 and H1975/OR cells by activating the Hippo pathway, which was key cancer signaling in association with cell proliferation, cell death, and cancer metastasis (Kulkarni et al., 2020). Co-treatment of Rg3 and osimertinib could promote expressions of the active components of the Hippo pathway, such as MST1/2 and LATS1/2, and depress the expression of the negative executor or effector. In addition, ginsenoside panaxatriol (GPT) (Wang et al., 2020) sensitized MDA-MB-231 PTX resistant (MB231-PR) cells to PTX through reducing the characteristics of stem cells, which was correlated with the IRAK1/NF-*κ*B and the ERK pathways. Co-treatment of GPT and PTX inhibited expressions of CSC related genes including octamer-binding transcription factor 4 (OCT4), sex determining region Y-box 2 (SOX2), NANOG, aldehyde dehydrogenase 1 (ALDH1), and CD44 gene expression, indicating that cell stemness-mediated invasion and tumor growth were inhibited by GPT and PTX. And IRAK1 and S100A7/8/9 formed a feedback loop to drive the malignancy of TNBC cells (Goh et al., 2017). The combination inhibited phosphorylation of IRAK1, P65 and ERK1/2, and promoted I*κ*B-alpha as well as inhibited S100A7/9 mRNA expression. In addition, the combination therapy also exerted an inhibitory effect on expressions of NF-*κ*B targeted genes including interleukin 6 (IL6), IL8, chemokine (C-X-C motif) ligand 1 (CXCL1), and chemokine (C-C motif) ligand 2 (CCL2). Thus, GPD could overcome PTX resistance by mediating stemness of tumor cells which resulted from an inhibition of the IRAK1/NF-*κ*B/ERK pathway.

## 5 Discussion

**FIGURE 3:**
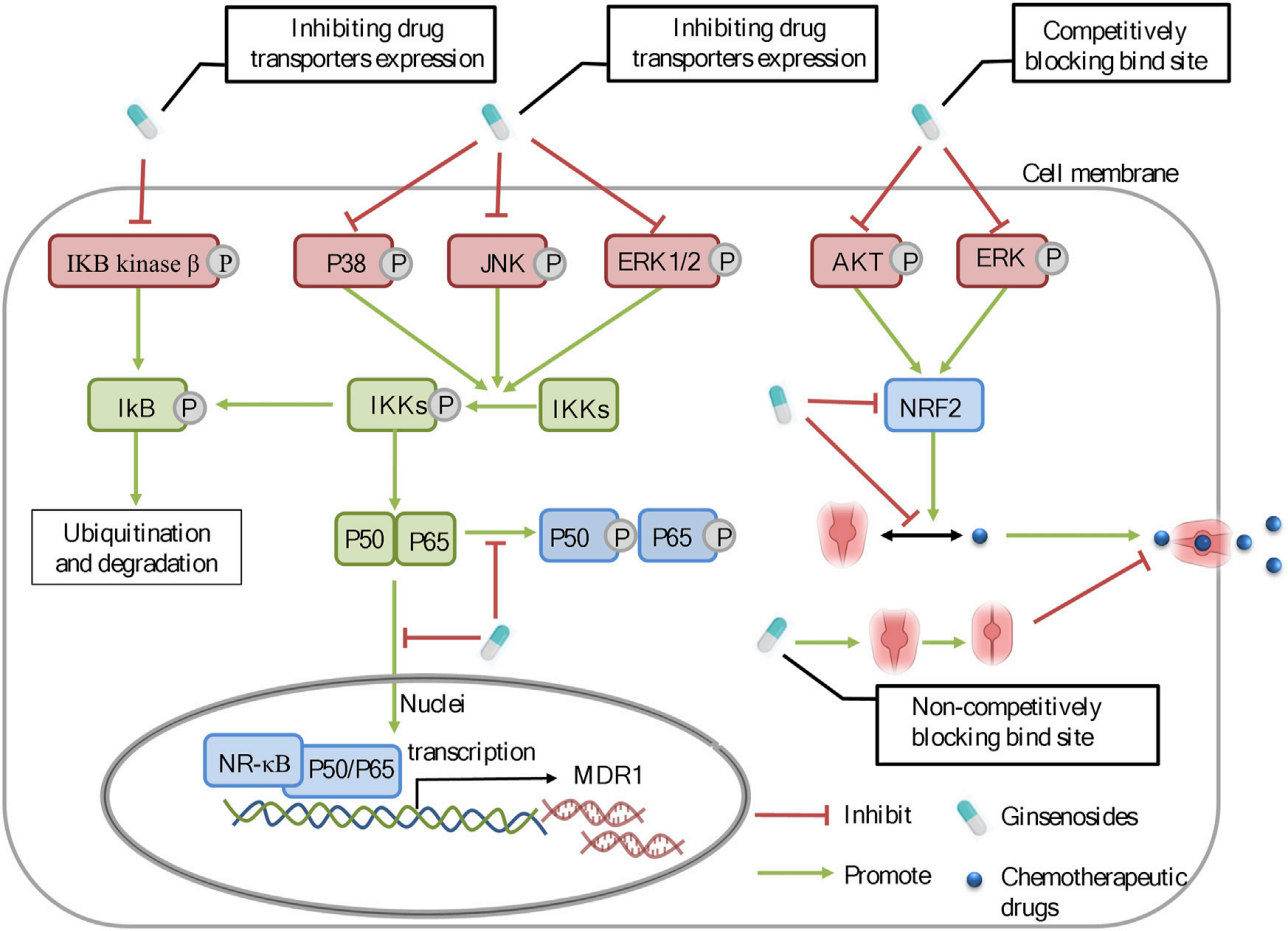
The molecular mechanisms of ginsenosides against chemoresistance through inhibiting the expression and function of drug transporters. Ginsenosides can regulate the ERK/NF-*κ*B and NRF2 signaling pathways to modulate the expression of drug transporters.

**FIGURE 4:**
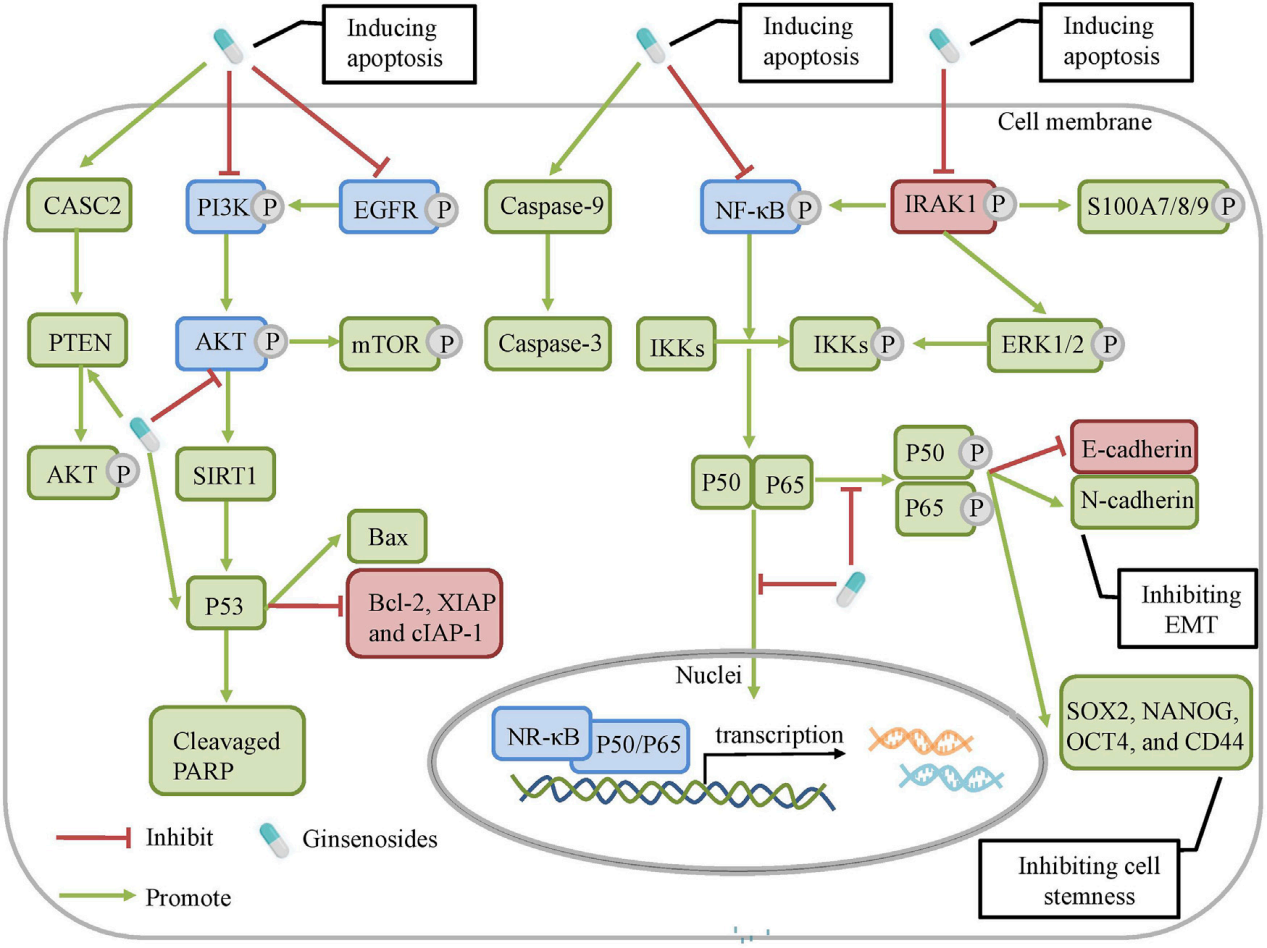
The molecular mechanisms of ginsenosides against chemoresistance. Ginsenosides can regulate NRF2/AKT, CASC2/PTEN, AKT/ SIRT1, EGFR/PI3K/AKT, PI3K/AKT/ m-TOR and NF-*κ* signaling pathways to modulate cell apoptosis, autophagy and tumor microenvironment.

Chemoresistance is a common phenomenon and the main barrier in cancer chemotherapy. Modern scientific studies have explained the causes of cancer chemoresistance from multi-perspectives and multi-dimensions, which has built a foundation for the development of chemoresistance-reversal agents. Currently, ginsenosides, originated from ginseng, have received much attention as potential preventive and therapeutic strategies against all kinds of tumors, particularly as supplements of chemotherapy in reversing chemoresistance. According to the previous reports, it is notable that Rg3 and Rh2, have the unique advantage over chemotherapeutic drugs and function as multi-targeted and multi-pathway chemoresistance-reversal agents. In this article, the inhibitory effect and mechanisms of ginsenosides on chemoresistance are summarized (Tables 1 and 2). The underlying mechanisms may be correlated with inhibition of drug transport proteins, modulation of autophagy, induction of apoptosis as well as alterations of TME (Figure 2). Further researches suggested that multiple signal pathways are involved in reversing chemoresistance, such as CASC2/PTEN/Akt, AKT/SIRT1, EGFR/PI3K/AKT, PI3K/AKT/mTOR, Nrf2/PI3K/AKT, and MAPK/NF-*κ*B pathways (Figures 3 and 4). Also, these studies revealed the structure-activity relationship of ginsenosides. The differences between the chemical structures of those ginsenosides might have quite a substantial influence on the mechanism of reversing chemoresistance. For instance, the P-gp inhibitory effect of 20 (S)-Rh2 and 20 (S)-PPD was more pronounced than that of 20 (R)-Rh2 and 20 (R)-PPD, respectively (Zhang et al., 2012b). Moreover, ginsenosides with higher molecular weights, including Rg3, PTG, Rb1, PPD12, and Rg5, were more likely to reverse chemoresistance through competitively blocking the binding site of P-gp whereas smaller molecules like 20(S)-Rh2 and aPPD in a non-competitively blocking manner (Table 2).

**TABLE 1:** Summary of the reversal effect of ginsenosides when combined with chemotherapeutic agents on chemoresistance in cancer treatment.

Cancer type	Drugs	Ginsenosides	Cell type	Effect	Mechanism	References
Esophageal cancer	Cisplatin	Rg3	Eca109/DDP	Reverse drug resistance	↓ p-gp expression	Li and Chen (2013)
				Inhibit tumor growth	↑cell cycle arrest in G1/S phase	
Colon cancer	Oxaliplatin	Rh2	LoVo/L-OHP, LoVo cell	Reverse drug resistance	↓ p-gp expression, Bcl-2	Ma et al. (2019)
				Inhibit proliferation	↑ Bax, caspase-3 and Smad4	
				Induce apoptosis		
Breast cancer	Adriamycin, Fluorouracil	Rh2	MCF-7/Adr, MCF-7	Reverse drug resistance	↓P-gp activity	Li and Chen (2013)
					↑Drug accumulation	
Breast cancer	Adriamycin	20(S)-Rh2	MCF-7/Adr, MCF-7	Reverse drug resistance	↓P-gp activity	Zhang et al., 2012c
				Induce apoptosis	↑Drug accumulation and distribution	
Breast cancer	Adriamycin	Rd	MCF-7/Adr	Reverse drug resistant	↓ MDR1 *via* ubiquitination-dependent protein degradation	Pokharel et al. (2010)
Breast cancer	Adriamycin	20(S)-Rh2	MCF-7/Adr	Reverse drug resistant	↓ ABCB1 expression, NF-*κ*B/MAPK pathway	Zhang et al., 2012a
Acute myelogenous leukemia	Daunorubicin	Protopanaxatriol ginsenosides, Protopanaxatriol ginsenosides, Rb1, Rb2, Rc, Rg1, Re	AM-2/D100, AM-2/DX100	Reverse drug resistance	↓Drugs inteact with P-gp	Choi et al. (2003)
					↑Drugs accumulation	
Human myeloid leukemia	Adriamycin	20(R)-Rg3	K562/ADM	Reverse drug resistant	↓P-gp	Gao et al. (2002)
				Induce apoptosis		
Fibroblast carcinoma	Vincristine, Doxorubicin	Rg3	KB V20C, P388/DOX	reverse drug resistant	↑Drugs toxicity, competitively blocking bind site	Kim et al. (2003)
				Increase life span of mice		
Acute promyelocytic leukemia	Vincristine	Rb1	HL60, HL60/VCR	Reverse drug resistant	↓ Drug bind to P-gp	Li et al. (2005)
					↑Cellular drug accumulation	
Breast Cancer	Adriamycin	20(S)-protopanoxadiol derivative	KB/VCR, MCF-7/ADM	Reverse drug resistant	↓ Drug bind to P-gp	Chen G et al. (2016)
Fibroblast carcinoma				Induce apoptosis	↑ABCB1 ATPase activation	
				Decrease the volume of tumor		
Ovarian cancer; Lung cancer	Docetaxel, Doxorubicin, Paclitaxel, Daunorubicin	Rg5	A2780/T, A549/T, A549	Reverse drug resistance	↓Drugs bind to P-gp, Nrf2/AKT pathways	Feng et al. (2020)
				Induce apoptosis	↑Cellular drug accumulation, ABCB1 ATPase	
				Increase cycle arrest in G2/M		
				Increase cytotoxicity		
Breast cancer	Digoxin, Fexofenadine, Etoposide	20(S)-ginsenoside Rh2	MCF-7/ADR, Caco-2 cells (HTB-37)	Reverse drug resistance	↓ P-gp, ATPase activity; change the binding sites	Zhao et al. (2009)
				Increase efficiency of anticancer		
Murine leukemia	Adriamycin	20(S)-Protopanaxadiol	P388, P388adr, MCF-7/ADR	Reverse multidrug resistance	↓P-gp activity	Zhao et al. (2009)
Breast cancer					↑ Cytotoxicity, cellular drug accumulation	
		aglycon 20(S)-protopanaxadiol		Induce apoptosis	↓ P-gp	Liu et al. (2004)
					↑ Caspase 3, 8, and 9, cytotoxicity	
Murine leukemia, Human fibroblast cancer	Adriamycin, Vincristine	20(S)-Rg3	P388/Adr, KBV20C	Reverse drug resistance	↓ Membrane fluidity, P-gp function	Kwon et al. (2008)
				Decrease the volume of tumor	↑Cytotoxicity	
Ovarian cancer	Actinomycin D, Paclitaxel, Doxorubicin	Rp1	OVCAR-8, NCI/ADR-RES, DXR	Reverse drug resistance	↓ MDR-1 protein expression and Src activition	Yun et al. (2013)
				Induce apoptosis	↑Redistribute lipid rafts, MDR-1 protein, DNA damage	
Lung adenocarcinoma	Cisplatin	Rg3	A549/DDP	Reverse drug resistance	↓MDR1, LRP, MRP expression	Zhang et al. (2002)
Lung cancer	Cisplatin	20(S)-Rg3	A549, A549/DDP	Reverse drug resistance	↓P-gp, MPR1, LPR1 expression	Liu C et al. (2018)
				Decrease tumor weight		
Colorectal Cancer	5-FU	Rh2	LoVo, LoVo/5-FU, HCT, HCT-8/5-FU	Reverse drug resistance	↓ MRP1, MDR1, LRP and GST expression, cyclin D1, CDK2, p-Rb, Bcl-2	Liu C et al. (2018)
				Induce apoptosis	↑ Cleaved-caspase 3	
				Increase cell cycle arrest: G0/G1 phase		
				Inhibit proliferation, migration and EMT		
Breast cancer	Mitoxantrone, Doxorubicin	Protopanaxadiol-containing ginsenosides (Rg3, Rh2, and PPD) and protopanaxatriol-containing ginsenosides (Rg1, Rh1, and PPT)	MCF-7, MCF-7/MX, MCF-7/Adr	Reverse drug resistance	↓ BCRP-associated vanadate sensitive ATPase activity	Jin et al. (2006)
				Inhibit drug efflux		
				Increase drug uptake		
Lung adenocarcinoma	Cisplatin	Rh2	A549/DDP	Inhibit tumor growth	↑Mitochondrial permeability, transmemtal potential, cytochrom C, caspase-3	Hu et al. (2010)
				Reverse drug resistance		
				Induce apoptosis		
Pancreatic cancer	Gemcitabine	Rg3	Panc-1, SW 1990, Panc-1/GEM, SW1990/GEM	Inhibit tumor growth	↑CASC2, PTEN	Zou et al. (2020)
				Induce apoptosis		
Colon cancer, Ovarian cancer, Lung cancer	Actinomycin D	Rp1	LS513, OVCAR8-DXR, A549-DXR	Induce apoptosis	↓SIRT1, AKT	Yun et al. (2020)
				Inhibit tumor growth	↑DNA damage, 53 acetylation, PARP clevage	
				Reverse drug resistance		
Pancreatic cancer	Erlotinib	Rg3	BxPC-3, AsPC-1	Inhibit proliferation	↓p-EGFR, p-PI3K, p-Akt	Jiang et al. (2017)
				Induce apoptosis	↑ Caspase-3, caspase-9, PARP cleavage	
				Increase tumor cells sensitivity		
				Decrease tumor growth		
Nasopharyngeal cancer	Paclitaxel	Rg1	SUNE1 cells, NP460	Inhibit cell viability	↓p62, Bcl-2	Li et al. (2019)
				Inhibit cell growth	↑LC3II, Bax,ROS, cycle arrest in S phase	
				Induce autophagy		
				Induce apoptosis		
Colon cancer	Docetaxel	Rg3	HCT116, SW620	Inhibit proliferation	↓DNA binding activity of NF-*κ*B, Bcl-2, XIAP and cIAP-1	Kim et al. (2009)
				Induce apoptosis	↑ Bax, caspase-3, caspase-9	
				Reverse drug resistance		
Prostate cancer	Docetaxel	Rg3	LNCaP, PC-3, DU145	Inhibit tumor growth	↓Activity of NF-*κ*B transcriptional activity, DNA binding activity of NF-*κ*B, Bcl-2, XIAP, cIAP-1	Kim et al. (2009)
				Reverse drug resistance	↑Cell cycle arrest: G0/G1 phase, Bax, caspase-3, caspase-9, cleaved PARP	
				Induce apoptosis		
Triple-negative breast cancer	Paclitaxel	Rg3	MDA-MB-231, MDA-MB453, BT-549	Inhibit cell viability	↓ NF-*κ*B activation, p65, Bcl-2	Yuan et al. (2017)
				Induce apoptosis	↑Bax, Caspase-3, cytotoxicity	
Human non-small cell lung cancer	Icotinib	20(S)-Rg3	PC-9, HCC827, PC-9R, HCC827R	Reverse drug resistance	↑ LC3-II, LC3II puncta formation, P62	Wang et al. (2019)
				Inhibit autophagy		
				Inhibit cell proliferation		
				Inhibit tumor growth		
Hepatocellular cancer	Doxorubicin	20(S)-Rg3	SK-Hep1, HepG2	Inhibite autophagy	↑ LC3 II, LC3II puncta formation, GFP-LC3 puncta formation, CHOP, P62	Kim et al. (2014)
				Induce cell death		
				Inhibit tumor weight and volume		
				Reverse drug resistance		
Ovarian cancer	Paclitaxel or cisplatin	Rb1, CK	SKOV-3, HEY A8 CSCs	Inhibit proliferation	↓Snail, Slug, E-cadherin, p-Akt, p-ERK1/2, Wnt/*β*-catenin signaling	Kala (2014)
				Inhibit CSC self-renewal	↑cytotoxicity, caspase-3, N-cadherin	
				Inhibit EMT		
Hypoxic lung cancer	Ciaplatin	Rg3	SPC-A1, H1299 cells	Inhibit EMT and stemness	↓Bcl-2 and survivin, Snail, N-cadherin, and Vimentin, NF-*κ*B DNA binding and p-p65, p65, p-IKK, IKK	Wang et al. (2018)
				Inhibit tumor growth	↑Caspase-3, -8, -9 and Bax,E-cadherin	
				Induce apoptosis		
				Inhibit tumor growth, weight and volume		
Non-small cell lung cancer	Osimertinib	Rg3	H1975	Reverse drug resistance	↓Cell stemness	Tan et al. (2020)
					↑ Hippo pathway	
Triple-negative breast cancer	Paclitaxel	Panaxatriol	MB231-PR; SUM159-PR	Induce apoptosis	↓BCL-2, MCL-1, p-IRAK1, P65, ERK1/2, S100A7, S100A9	Wang et al. (2020)
				Inhibits cancer stemness	↑Bax, I*κ*B-alpha, cytotoxicity	
				Inhibit cell growth		

**TABLE 2:** Summary of molecular mechanisms of main ginsenosides against chemo-resistance.

Ginsenosides	Inhibiting the drug transporters expression	Competitively blocking the binding sites of P-gp	Non-competitively blocking the binding sites of P-gp	Inhibiting lipid efflux and lipid raft	Inducing apoptosis	Modulating autophagy	Altering TME
Rg3	+	+	—	+	+	—	+
20(S)-Rg3	+	+	—	—	—	+	—
Rh2	+	—	—	—	+	—	—
20(R)-Rh2	—	—	+	—	—	—	—
PPD	+	+	—	—	—	—	—
PPD12	—	+	—	—	—	—	—
aPPD	—	—	+	—	—	—	—
PTG	—	+	—	—	—	—	—
Rb1	—	+	—	—	—	—	+
Rg5	—	+	—	—	—	—	—
Rd	+	—	—	—	—	—	—
Rp1	—	—	—	—	+	—	—
Rg1	+	—	—	+	+	+	—
GPT	—	—	—	—	—	—	+

Substantial evidence *in vitro* and *in vivo* reveal the benefits of the combination of ginsenosides and chemotherapy on chemoresistance. However, according to the current studies in this field, these findings also have certain limitations. Firstly, ginsenosides are characterized by low oral bioavailability caused by poor oral absorption, usually <5% in the rodent model (Xu et al., 2003). Therefore, various drug delivery systems and routes should be taken to improve oral bioavailability or exert a novel effect (Hong et al., 2019). Such as the multifunctional liposome system, ginsenosides functioned as not only the chemotherapy adjuvant to exert their inherent anticancer activity, but also the functional membrane material to promote effectiveness in cancer therapy in this system. Furthermore, although studies showed that poor oral bioavailability and absorption, as well as the potency of the anti-tumor effects of ginsenosides, were related to their molecular weights and structure (Zhang et al., 2012b), the structure-activity relationship of ginsenosides has not been completely illuminated. Hence, some measures, such as chemical synthesis and structural modification should be taken to improve the chemoresistance reversal activity of ginsenosides. Secondly, there are a high proportion of studies on classical mechanisms of ginsenosides-induced chemoresistance reversal, mainly involving P-gp and apoptosis mediated chemoresistance, whereas a low proportion of studies on non-classical mechanisms, which are attributed to regulation of GST-*π*, Topo II, CSCs, et al. Therefore, the metabonomics and molecular docking techniques are needed in the further studies. Also, the quartz crystal microbalance (QCM) (Zhou et al., 2012), a novel monitor method is a preferable choice compared to MTT to real-time monitor the chemotherapy induced death process of tumor cells. Finally, most of the current studies focus on the effect of ginsenosides on chemoresistance through animal models and cells, but there are insufficient clinical researches of such benefits. Thus, large sample-sized, randomized, controlled, and clinical studies should be carried out to investigate their actual effects on chemoresistance, setting a higher level of evidence for the therapeutic effects of ginsenosides.

This article systematically summarizes the therapeutic potential of ginsenosides in reversing chemoresistance *in vivo* and *in vitro* and illuminates the mechanisms by which ginsenosides alleviate chemoresistance. The underlying molecular mechanism can be partially elucidated by the roles of the expression and function of P-gp, MDR1, BCRP, LRP, apoptosis, autophagy, and microenvironment as well as CASC2/PTEN/AKT, AKT/SIRT1, EGFR/PI3K/AKT, PI3K/AKT/mTOR and MAPK/NF-*κ*B signaling pathways. Given that ginsenosides can also reduce side effects caused by chemotherapy and elicit anti-cancer activities and little toxicity, they have demonstrated promises as an indispensable strategy for reversing chemoresistance. We look forward to further revealing their reversal mechanisms and widespread clinical applications.

## Glossary

ABC: ATP-binding cassette
ActD: actinomycin D
ADM: Adriamycin
BCRP
Breast cancer resistance protein
CK: Compound K; CQ, Chloroquine
CSCs: Cancer stem cells
DDP: Cisplatin
DOX: Doxorubicin
EGFR: Epidermal growth factor receptor
EGFR-TKI: Epidermal growth factor receptor-tyrosine kinase inhibitor
EMT: Epithelial-mesenchymal transition
FU: Fluorouracil
GEM: Gemcitabine
GST: Glutathione-S-transferase
HCQ: Hydroxychloroquine
IL: Interleukin; L-OHP, Oxaliplatin
LRP: Lung resistance-related protein
MAPK: Mitogen-activated protein kinase
MDR1: Multi-drug resistance 1
MRP: Multidrug resistance-related protein
m-TOR: Mammalian target of rapamycin
MX: Mitoxantrone
NF-κB: Nuclear factor-κb
NRF2: Nuclear factor erythroid-2 related factor 2
P-gp: P-glycoprotein
PI3K: Phosphatidylinositol 3-kinase
PKC: protein kinase C
PPD: Protopanoxadiol
PPT: Protopanaxatriol
PTEN: protein tyrosine phosphatase gene
PTG: Protropanaxatriol ginsenosides
Rho123: Rhodamine 123
SIRT1: Sirtuin1
TME: Tumor microenvironment
TNBC: Triple-negative breast cancer
TOPOII: Topoisomerase II
TXT: Docetaxel
VCR: Vincristine
Vp: Verapamil
